# The Neurology of Death and the Dying Brain: A Pictorial Essay

**DOI:** 10.3389/fneur.2020.00736

**Published:** 2020-07-21

**Authors:** Daniel Kondziella

**Affiliations:** ^1^Department of Neurology, Rigshospitalet, Copenhagen University Hospital, Copenhagen, Denmark; ^2^Department of Clinical Medicine, University of Copenhagen, Copenhagen, Denmark

**Keywords:** brain death, cardiac arrest, coma, consciousness, migraine aura, near-death experiences, organ donation, resuscitation

## Abstract

As neurologists earn their living with the preservation and restoration of brain function, they are also well-positioned to address the science behind the transition from life to death. This essay in pictures highlights areas of neurological expertise needed for brain death determination; shows pitfalls to avoid during the clinical examination and interpretation of confirmatory laboratory tests in brain death protocols; illustrates the great variability of brain death legislations around the world; discusses arguments for the implementation of donation after circulatory death (DCD); points to unresolved questions related to DCD and the time between cardiac standstill and organ procurement (“hands-off period”); provides an overview of the epidemiology and semiology of near-death experiences, including their importance for religion, literature, and the visual arts; suggests biological mechanisms for near-death experiences such as dysfunction of temporoparietal cortex, N-methyl-D-aspartate receptor antagonism, migraine aura, and rapid eye movement sleep; hypothesizes that thanatosis (aka. death-feigning, a common behavioral trait in the animal kingdom) represents the evolutionary origin of near-death experiences; and speculates about the future implications of recent attempts of brain resuscitation in an animal model. The aim is to provide the reader with a thorough understanding that the boundaries within the neurology of death and the dying brain are being pushed just like everywhere else in the clinical neurosciences.

## Introduction

While the protection and repair of brain function is the *raison d'être* for the practicing neurologist, neurological expertise can also be applied to the transition from life to death.

The most obvious arena where this expertise is needed concerns the determination of brain death in a potential organ donor (1). In addition, donation after circulatory death (DCD) is increasingly practiced when brain-injured patients are deemed unlikely to enter brain death before withdrawal of life-sustaining therapy (2). In these cases, neurologists are typically consulted to confirm that meaningful recovery of brain function is futile, which requires proficiency in neuro-prognostication (3, 4).

Further, knowledge about the cellular processes in the brain taking place after circulatory arrest is important to determine how much time should pass between cardiac standstill and organ procurement (3, 5). This is an important ethical problem because the amount of time elapsed is positively correlated with the certainty of lost brain function but negatively with the tissue quality of organ transplants (3, 5–7). Recent attempts of brain resuscitation in an animal model add another layer of complexity (8).

In addition, neurology may offer a window to understand near-death experiences (NDE) which are empirically testable despite their mystic flavor. Proposed biological mechanisms underlying NDE include temporoparietal lesions (9), N-methyl-D-aspartate receptor (NMDAR) hypofunction (10), rapid eye movement (REM) sleep intrusion (11), and migraine aura (12), all of which are familiar to neurologists.

This paper highlights salient aspects of death and the dying brain, aiming to provide busy neurologists with a rapidly accessible overview including graphic information. The work is based on a lecture that was due for presentation at a conference meeting canceled during the COVID-19 pandemic in 2020. As such, its intention is to share the author's personal view in an entertaining manner rather than to provide an exhaustive and strictly balanced review of the literature.

## An Historical Overview of Organ Donation After Brain Death and Circulatory Death

**Figure 1:**
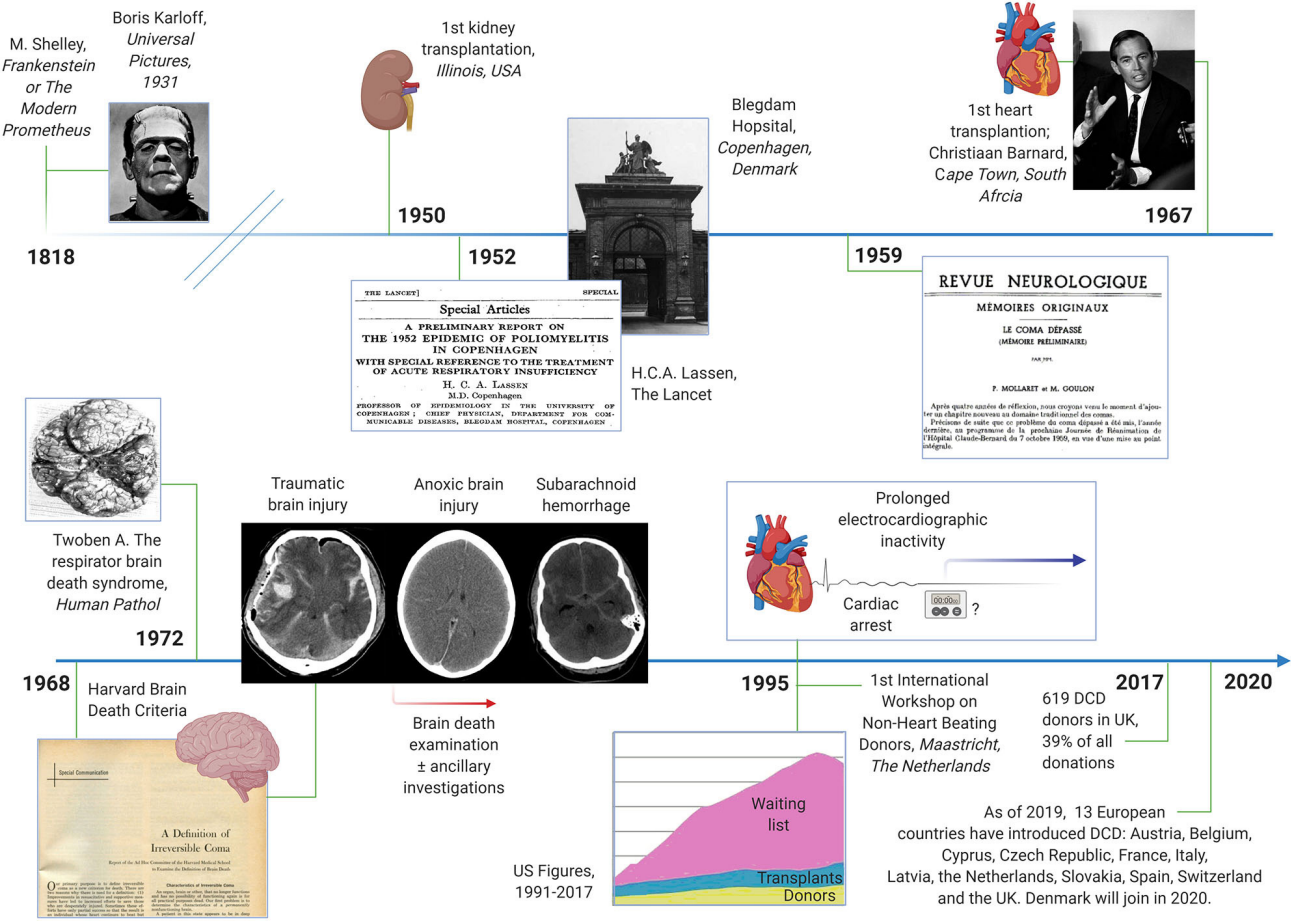
Timeline of historical events related to brain death and organ donation after brain death and circulatory death. Milestones include the first kidney and heart transplantations, the advent of artificial ventilation and the intensive care unit, the Harvard brain death criteria and the introduction of donation after circulatory death (DCD). Images in the public domain; courtesy of Wikipedia.

The notion that organs from deceased people can be used to serve the living is many centuries old, the most prominent example being Mary Shelley's *Frankenstein or The Modern Prometheus* from 1818 (Figure 1). The mid-20th century saw the first successful organ transplantations. In 1950, a kidney was transplanted at the Little Company of Mary Hospital in Evergreen Park, Illinois, USA; and 17 years later, at the Groote Schuur Hospital in Cape Town, South Africa, Christiaan Barnard performed the first heart transplantation in a man who recovered consciousness post-surgery (although the patient died 18 days later of pneumonia). The rise of intensive care medicine around the same time was key to the concept of brain death. The Blegdams Hospital (now defunct) in Copenhagen is typically credited for having established the first intensive care unit in the world in response to the 1952 poliomyelitis outbreak in Denmark's capital (13). H.C.A. Lassen reported in *The Lancet* that artificial ventilation was able to reduce the mortality from polio by half (14). Soon thereafter, it was recognized that the bodies of people, except their brains, could be kept functioning for a prolonged period given intensive care management. In 1959, “after 4 years of thinking,” Frenchmen Mollaret and Goulon termed this condition *coma dépassé* (literally, “a state beyond coma”) (15). The Harvard brain death criteria were published barely a decade later (16). Although the criteria have been revised several times since, they captured the essential features of brain death. In contrast to present day standards, however, the absence of spinal reflexes was a prerequisite for the diagnosis (16). Pathologists noted in the early 1970'ies that corpses which had been kept on prolonged intensive care support had brains that were “swollen, mottled gray-red, and extremely soft, at times diffluent; at autopsy, very often the mass of cerebrum flattens, detaches, and pours away from the base of the skull,” the so-called respirator brain death syndrome (17).

Over the subsequent years, organ donation after brain death became the standard. Organs for transplantation were no longer obtained from non-heart beating donors (NHBD), but most organ donors were now recruited after traumatic brain injury, cardiac arrest, and subarachnoid hemorrhage. Yet in the 1990ies it became clear that patients in need of organs progressively outnumbered donors and transplants^1^. Additional methods to increase the rate of organ donations were needed. In 1995, donation after circulatory death (DCD) was therefore (re-) introduced during the First International Workshop on NHBD, organized in Maastricht, the Netherlands (18). The idea was that DCD would help to reduce the shortage of organs by expanding the donor pool (19). DCD criteria have been modified several times since. In Europe, DCD is practiced most often in the Netherlands and in the UK. In 2017, 619 DCD donors were counted in the UK, roughly 40% of all deceased organ donors that year^2^. Given that many other countries are expected to introduce DCD programs, e.g., Denmark in 2020, the impact of DCD is destined to increase in the future.

## Brain Death Criteria

**Figure 2:**
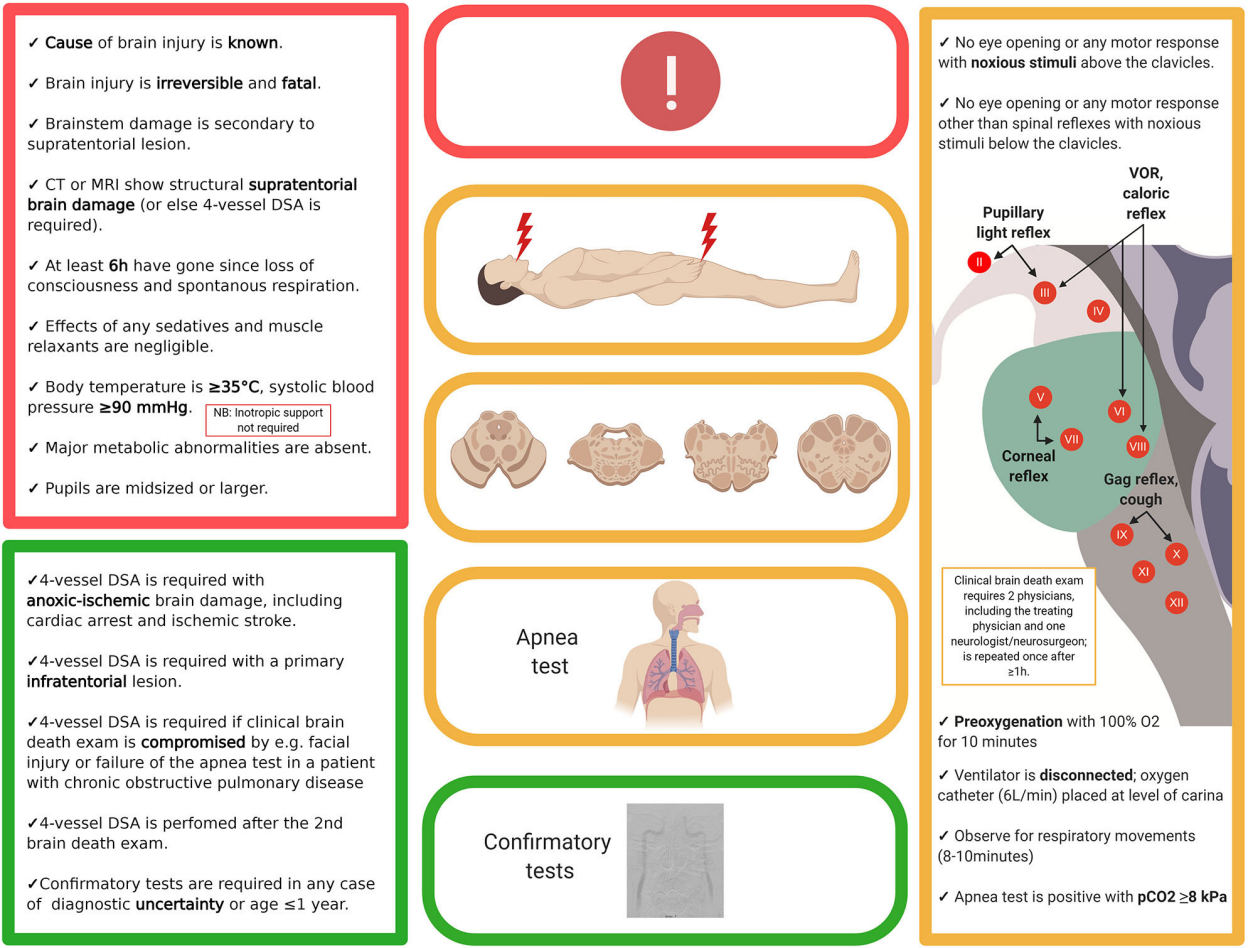
Example of a brain death protocol (Denmark). While brain death protocols vary widely, most follow the outlined three-step approach, i.e., documentation that the prerequisites for a brain death protocol are fulfilled (red), clinical examination (yellow) and—if required—confirmation by ancillary testing (green). The present example is in accordance with the legislation in Denmark; thus, readers are advised to check with their own local or national protocols. DSA, digital subtraction angiography; EEG, electroencephalography.

**Figure 3:**
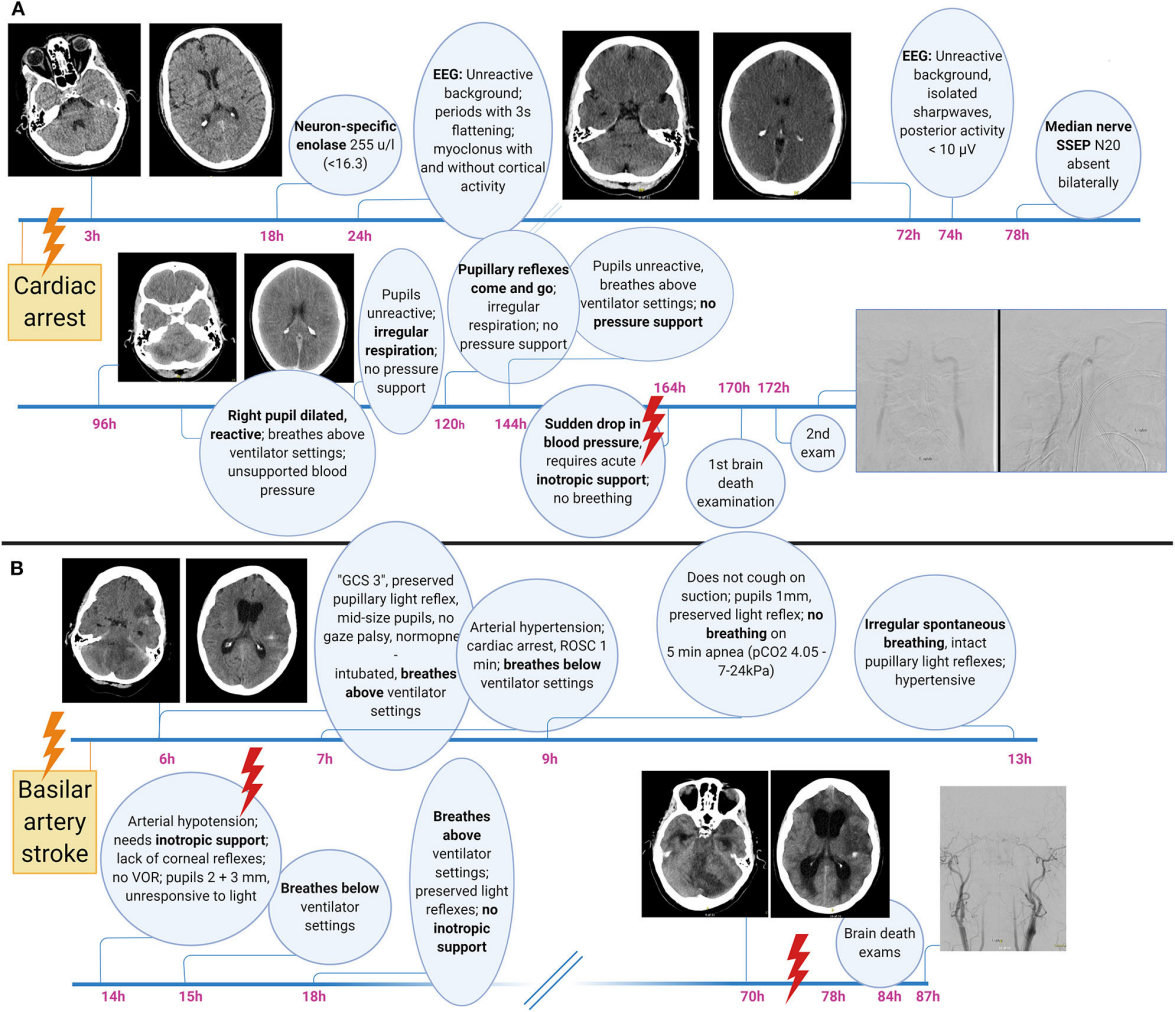
Illustrative brain death case examples related to global bran injury **(A)** and primary infratentorial brain damage **(B)**. Note the waxing and waning of brainstem function including pupillary light reflexes, the prolonged time from primary injury to clinical incarceration, heralded by the sudden onset of hypotension requiring inotropic support, as well as the development of secondary supratentorial damage including obstructive hydrocephalus after a primary infratentorial (i.e., brainstem) injury. GCS, Glasgow Coma Scale; SSEP, somatosensory evoked potentials.

The determination of brain death follows a three-step approach, with the third step (confirmatory laboratory investigations) being either unnecessary, optional, or mandatory. In analogy to a traffic light, these three steps can be thought of as the red, the amber and the green phases (Figure 2, showing an example of a brain death protocol). The red phase implies to the clinician to pause and to confirm that the prerequisites for a brain death protocol are fulfilled. These prerequisites cover two aspects. The first is the unconditioned certainty that the brain disorder faced is irreversible and fatal, which requires that the cause of brain injury is known and well-understood. Restated, people with an unknown cause of brain injury cannot be declared brain-dead. The second aspect is to ensure that the circumstances to assess brain death are optimal, i.e., that physiological parameters are within an acceptable range and that confounders such as sedation and muscle relaxants are ruled out. Hence, it is advisable to allow 5–7 times of any drug's elimination half-life to pass prior to the clinical examination and, if needed, to prove lack of neuromuscular blockade by revealing muscle twitches following electrical stimulation of the ulnar nerve (1). In the amber phase, the clinician proceeds to the clinical examination, which has three goals. The first is to document absence of signal transmission between the brain and the spinal cord; i.e., noxious stimuli applied to the head must not evoke a response in the remainder of the body, and noxious stimuli applied to the body must not evoke a response above the level of the foramen magnum (for caveats related to this rule, see below). The second goal is to confirm lack of brainstem reflexes, and the third is to confirm failure of hypercapnia to trigger the respiratory brainstem centers (apnea test). Depending on the clinical situation and the legislation, a variety of confirmatory tests may be used in the green phase to confirm the clinical examination. These tests are either based on the documentation of absent intracranial blood flow (e.g., digital subtraction angiography, computed tomography angiography, transcranial doppler, positron emission tomography, nuclear scintigraphy) or lack of electrical brain activity (e.g., EEG, evoked potentials). Although legislations of most countries require ancillary investigations under certain circumstances, it has been argued that these investigations often lead to confusing results (see below) and should be reserved for situations in which an apnea test cannot be performed because of hemodynamic instability, poor oxygenation, or chronic carbon dioxide retention (e.g., in patients with chronic obstructive pulmonary disease) or in which facial injury renders full examination of brainstem reflexes impossible (20). Others, by contrast, have pointed out the value of ancillary investigations in primarily infratentorial brain lesions (21). Illustrative case examples of developing brain death, one related to global anoxic-ischemic injury and the other to basilar artery stroke, are shown in Figure 3.

Besides a thorough knowledge of the legislation and of the details of local brain death protocols, neurologists performing brain death examinations must be aware of potential pitfalls. These relate to failures to adhere to the brain death protocol, including premature assessment of patients, misinterpretation of certain clinical signs as signaling preserved brain function and a lack of knowledge about the pathophysiological processes during the dying process that can confound the interpretation of ancillary investigations.

First, failure to adhere to brain death protocols may happen when clinicians deviate from the rule that the cause of irreversible brain damage has to be known or when they overlook confounders, which has occurred in a number of reports related to “brain death mimics” associated with, for instance, intoxications [e.g., baclofen (22)] and neuromuscular disorders [e.g., Guillain Barre syndrome (22)]. Hence, a brain death protocol should be stopped immediately when it is evident that repeated neuroimaging reveals lack of major cerebral injury (1).

Second, certain clinical signs may be misinterpreted as reflecting preserved brain function. For instance, both pupillary constriction and dilation may occur after brain death due to rudimentary contractility of the iris muscles. It is normal for pupils to dilate during the final stage of the dying process, when (or better: if) catecholamines are released into the blood stream, which over the ensuing hours will result in slight pupillary constriction when catecholamine molecules are metabolized. Further, pupillary dilatation as a spinal sympathetic reflex may occur with skin incision and cross-clamping during organ procurement (23). A similar reflex mechanism has been suggested in a notable case of a confirmed brain death in which the corpse repeatedly showed slow left eye opening in response to noxious stimulation of the ipsilateral nipple (but no other area), most likely due to sympathetically mediated activity of the superior tarsal muscle (aka. Muller's muscle) (24). While these clinical signs only rarely will lead to diagnostic confusion, awareness of spinal motor reflexes is crucial because these may become very evident. It is important to note that spinal motor reflexes tend to occur a few hours after declaration of brain death, the reason being that brain death equals a high upper cervical transection, which will result in spinal shock and muscular atonia that may transform over the ensuing hours into increased spinal activity with loss of supranuclear inhibitory control. Spinally mediated movements may result in distinct motor patterns that may mimic decerebrate rigidity (although evidently, this motor pattern is only induced by noxious stimuli below the foramen magnum) or reflect primitive reflexes such as the grip reflex (25). Tonic neck reflexes may be preserved as well, the most dramatic example being the Lazarus' sign in which intricate sequences of movements may develop over many seconds, including flexion of both arms to the chest, adduction of shoulders, hands raising up to the neck, with fingers held in dystonic postures, followed by crossing of the hands that finally move down to the bed again (26). The Lazarus' sign should not be confused with the Lazarus' phenomenon which refers to autoresuscitation and which is incompatible with brain death (27).

Third, ignorance of the pathophysiological processes during the dying process may lead to erroneous interpretation of ancillary investigations. Most flawed conclusions can be avoided when remembering that the transition from (rudimentarily preserved) cerebral activity to (a clinical diagnosis of) brain death is not sharply defined but occurs typically over several minutes or even hours. The clinical examination relies on the assessment of the brainstem, which explains why islands of electrically functioning cortical neurons may give rise to residual EEG activity (28) and why rudimentary intracranial perfusion may be revealed by digital subtraction angiography for a limited time after a correctly performed clinical brain death examination. The author knows of a case in which organ procurement was stopped because of doubts related to spinal reflexes occurring several hours (sic) after a correct clinical brain death protocol, followed by a digital subtraction angiography revealing very weak filling of the proximal intracranial vasculature. In this regard, it is important to be aware that most brain death protocols, including that presented in Figure 2, do not require inotropic support of blood pressure as a prerequisite for brain death. Restated, it is occasionally possible to declare brain death although the corpse supports its own blood pressure and although blood pressure control is mediated via the caudal ventrolateral medulla which is part of the brainstem (29). In this situation, it should therefore come as no surprise that digital subtraction angiography may show rudimentary intracranial filling of proximal vessels. Thus, as a rule of thumb, whenever documentation of complete absence of intracranial blood flow is mandatory (e.g., in Denmark following anoxic, but not traumatic, brain injury, Figure 2), neurologists should refrain from starting a brain death protocol until inotropic blood pressure support has become necessary and the patient is polyuric (1).

**Figure 4:**
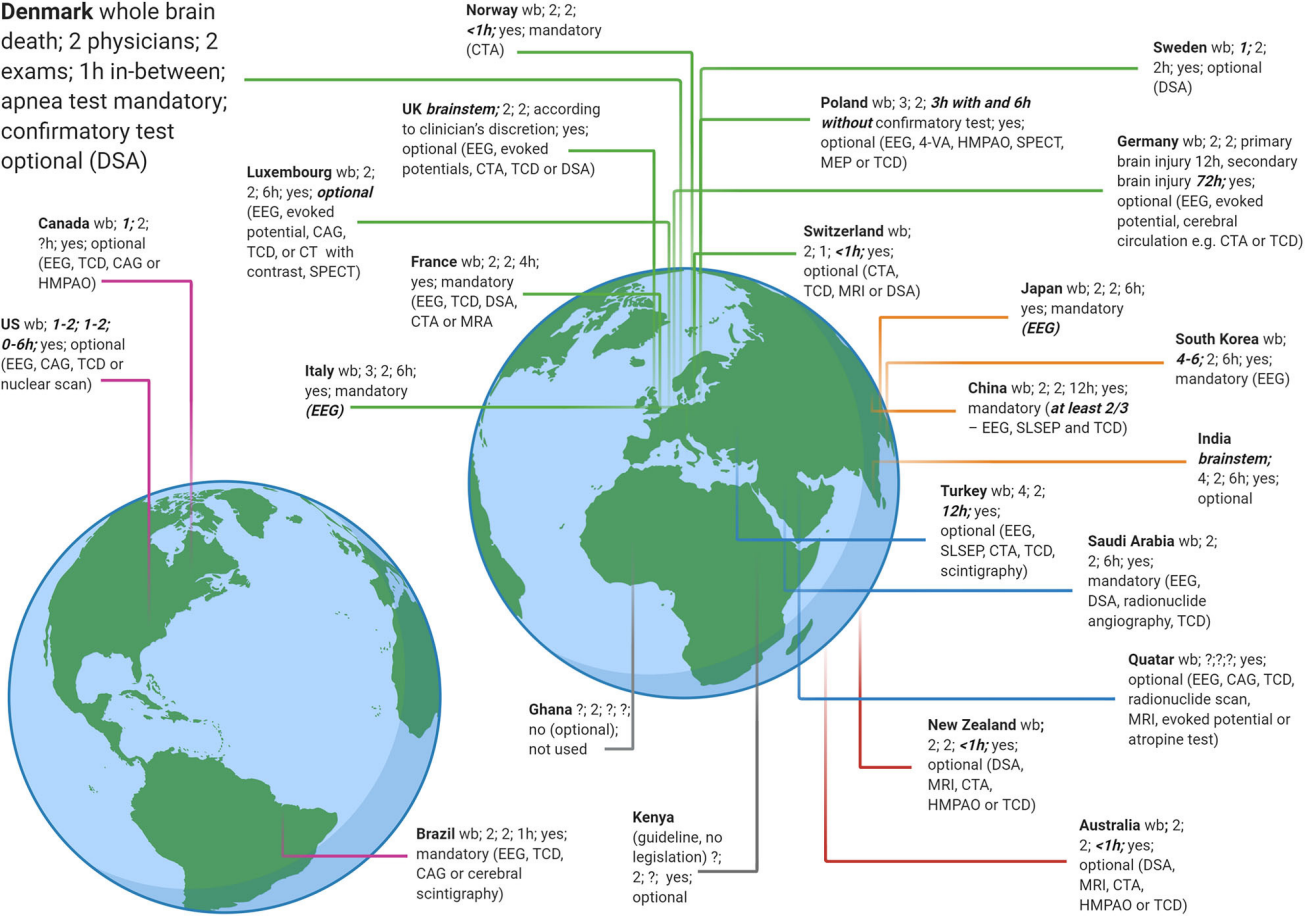
Brain death criteria from 24 countries. A recent study (30) revealed as many different brain death legislations as countries sampled. Data adapted with permission from (30). A few criteria that deviate from most countries are highlighted (somewhat arbitrarily) in bold and italics, e.g., up to 6 physicians are required to take part in a brain death protocol in South Korea but only 1 in Sweden. 4-VA, four vessel angiography; CAG, cerebral angiography; CTA, computed tomography angiography; DSA, digital subtraction angiography; EEG, electroencephalography; HMPAO, hexa-methyl-propylene-amine oxime; MEP, motor evoked potential; MRA, magnetic resonance imaging angiography; MRI, magnetic resonance imaging; SLSEP, short-latency somatosensory evoked potential; SPECT, Single-photon emission computed tomography; TCD, transcranial doppler; wb, guidelines follow the concept of whole brain death (in contrast to brainstem death as in the UK and India).

Although brain death protocols must be strictly adhered to, it is important to keep in mind that they vary widely in detail from country to country (30, 31) (Figure 4). While these protocols almost universally follow the three-step approach as outlined earlier, a recent study (30) found as many different protocols as countries surveyed (*n* = 24). For instance, whereas 1 h must pass between the two clinical brain death examinations in Brazil, the time interval in Luxembourg is 6 h and in Germany between 12 and 72 h. In Sweden and Canada, legislation requires only 1 physician to perform the clinical examination, but at least 4 physicians are needed in South Korea. Digital subtraction angiography is needed in Denmark in certain situations, whereas in Italy and Japan EEG is mandatory (30). Even within the same country, e.g., the USA, guidelines and practices may vary according to geography (32). Also, while in most countries a patient is declared brain dead when clinical examination and confirmatory tests (if required) indicate irreversible loss of activity of the entire brain (“whole brain death”), the UK and India, by contrast, have implemented the concept of brainstem death, i.e., evidence of the irreversible loss of brainstem activity is sufficient (33, 34).

## Donation After Circulatory Death (DCD)

**Figure 5:**
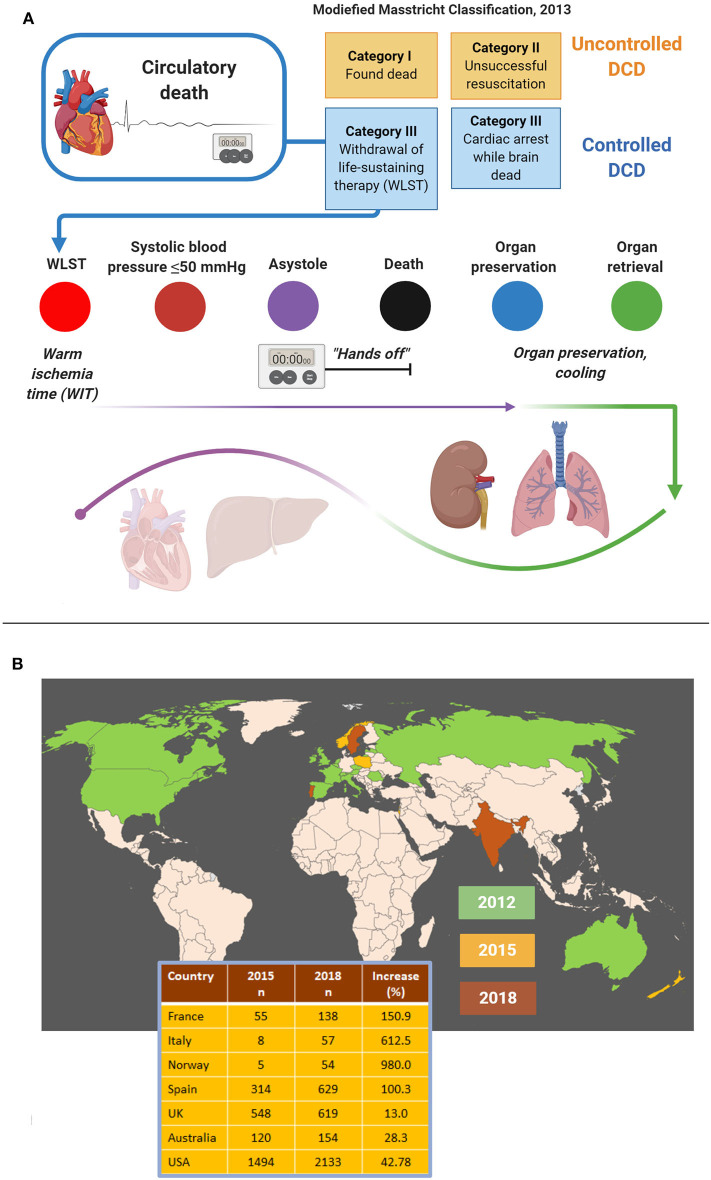
Procedural aspects **(A)** and worldwide figures **(B)** concerning donation after circulatory death (DCD). DCD is most commonly practiced as controlled DCD following withdrawal of life-sustaining therapy. Time periods that can be distinguished include the warm ischemia time, the “hands-off period” and the cooling period. While lungs and kidneys are more robust to the detrimental effects of warm ischemia, hearts and livers are procured much less often because of major tissue damage **(A)**. Still, DCD is on the rise globally, both in terms of the number of countries practicing DCD and increasing donor cases **(B)**. Data adapted with permission from (30).

According to the Maastricht criteria (18, 19), DCD is classified into uncontrolled DCD and controlled DCD (Figure 5). Uncontrolled DCD refers to organ retrieval following unexpected cardiac arrest from which the patient cannot or should not be resuscitated. In contrast, controlled DCD takes place in a planned setting where the decision of withdrawal of life-sustaining treatment is thought to be in the best interest of the patient given the expected poor neurological outcome. Hence, resuscitation is only attempted in uncontrolled DCD patients, but both settings involve a non-intervention period (“hands-off period”) to rule out autoresuscitation (35). Loss of cardio-pulmonary function is documented by e.g., echocardiography, electrocardiogram, and invasive monitoring of pulse and blood pressure (35). The period of non-intervention is variable and extends from seconds to several minutes (35–37). Circulation is then restored, involving cannulation and extracorporeal membrane oxygenation, and organs are explanted. Lungs and kidneys are more robust to the detrimental effects of prolonged warm ischemia time than the liver and the heart; but both the latter organs are increasingly transplanted as well (6, 7).

The number of countries that practice DCD is rapidly increasing, although the share of DCD in terms of organs transplanted is still low compared to organs from brain-dead donors. In 2019, DCD represented the major source for organs only in the Netherlands (30). Like brain death protocols, DCD protocols vary considerably from country to country. Guidelines require documentation of the absence of circulation and spontaneous respiration either by electrical asystole on ECG and/or lack of arterial pulsations demonstrated by arterial pressure monitoring. Other criteria such as heart auscultation, absence of a palpable pulse, skin perfusion, immobility, unresponsiveness to verbal or tactile stimuli, and loss of brainstem reflexes, vary between countries. As stated earlier, before the patient can be declared dead guidelines usually require a “hands-off period,” during which physicians must not intervene, to rule out autoresuscitation. The length of this period varies from 2 min in the UK to 10 min in Spain or is unspecified (Australia, New Zealand) (30), but a recent survey showed that 10 out of 64 (16%) European centers immediately retrieve organs after a flatline ECG (38).

## Cellular Mechanisms In The Dying Brain

**Figure 6:**
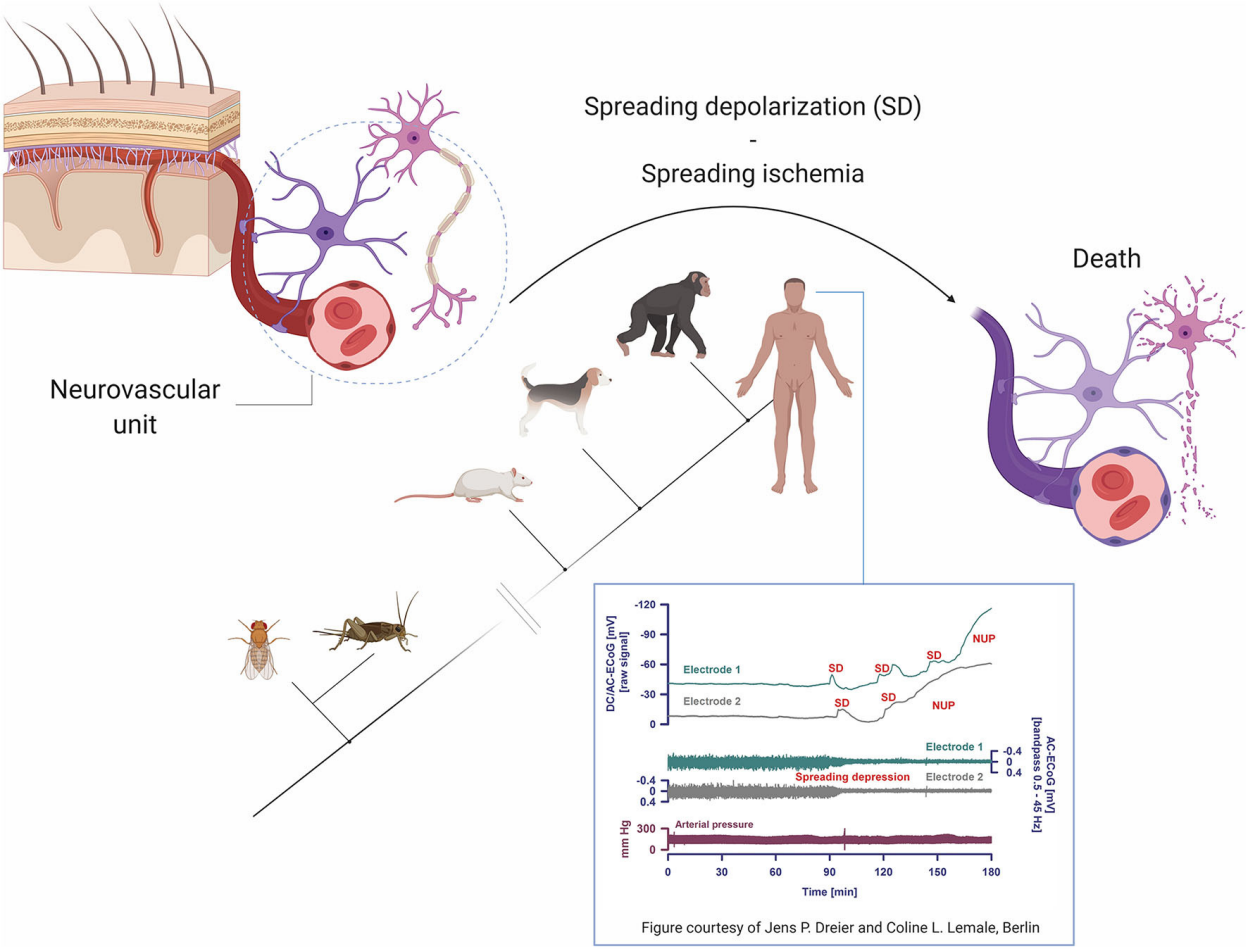
Pathophysiological mechanisms and phylogenetic aspects of spreading depolarizations (SD). Terminal SD involves impaired neurovascular coupling and is the biological hallmark of the dying neuron and brain death. Of note, terminal SD is a phylogenetically highly preserved mechanism and must also have occurred in the last common ancestor of humans and insects for over 500 million years ago. Figure inset shows intracranial recordings from a patient with subarachnoid hemorrhage at the time of brain death, reprinted with permission from (12) and courtesy of Jens P. Dreier and Coline L. Lemale, Berlin. Note that while arterial pressure remains stable (i.e., the heart is still functioning), terminal SD evolve into negative ultraslow potentials (NUP) and spreading depressions, marking the transformation to brain death. DC/AC ECoG, direct current/alternating current electrocorticography.

Neurovascular coupling is fundamental to the physiology of the living and the dying brain. The term refers to interactions within the “neurovascular unit,” which consists of neurons, astrocytes and vascular cells, including the blood-brain barrier (Figure 6). Briefly, neuronal activation is accompanied by increased cerebral blood flow and modestly increased cerebral metabolic rate for oxygen, leading to functional hyperemia and energy supply (39). Neurovascular coupling thus reflects the close temporal and regional connection between neuronal activity and cerebral blood flow (39–42). Impairment of neurovascular coupling is associated with the development of spreading depolarizations (SD), including terminal SDs and spreading ischemia which are the final cellular mechanisms in the dying cerebral cortex (43–45).

Although terminal SDs without repolarization mark the transition from life to (cerebral) death, there are three accompanying events that occur always, albeit not automatically in the same sequence: loss of respiration, blood flow, and spontaneous electrocorticographic activity (44–46). With circulatory death, the most common scenario, arrest of systemic circulation, respiration, and electrocorticographic activity occurs more or less at the same time, while terminal SDs follow the complete arrest of electrocorticographic activity after 13–266 s (44, 45).

The sequence of events is different in brain death. At the neuronal level, toxic cellular changes include gradually increasing intracellular calcium and extracellular potassium concentrations, decreasing extracellular pH, exhaustion of adenosine triphosphate (ATP) stores and failure of ATP-dependent membrane pumps such as the Na,K-ATPase which becomes unable to replenish leaking ions (47–52). Vesicular release of various transmitters, including glutamate and γ-aminobutyric acid, may lead to an incoherent, massive increase in miniature excitatory and inhibitory postsynaptic potentials, replacing normal postsynaptic potentials and disrupting regular neuronal interactions (53–55). Neuronal hyperpolarization turns into neuronal depolarization. Clinically, following the onset of dilated pupils and loss of brainstem reflexes, indicating imminent brain death, electrocorticography may reveal terminal clusters of SDs (inset, Figure 6), propagating from one electrode to the next and indicating terminal depolarization. Although the negative ultraslow potentials signal irreversible neuronal death, the arterial blood pressure remains stable (46). In the intensive care setting, loss of circulation and respiration, indicating cessation of cardiopulmonary activity, occurs typically first after terminal extubation.

Virtually all animals with a central nervous system, including insects (56) and humans (44, 45), undergo terminal SD at the end of their life. Using phylogenetic bracketing (a method to infer the probability of unknown traits in organisms based on their location in a phylogenetic tree), we can conclude that terminal SDs must have occurred in the last universal common ancestor of insects and humans as well. In other words, terminal SDs must have been the final neuronal pathway to death for probably as long as there has been life with a nervous system on Earth, i.e., at least since the “Cambrian explosion” about 520 to 560 million years ago (57, 58).

## Near-Death Experience—Phenomenology, Epidemiology, and Biological Mechanisms

**Figure 7:**
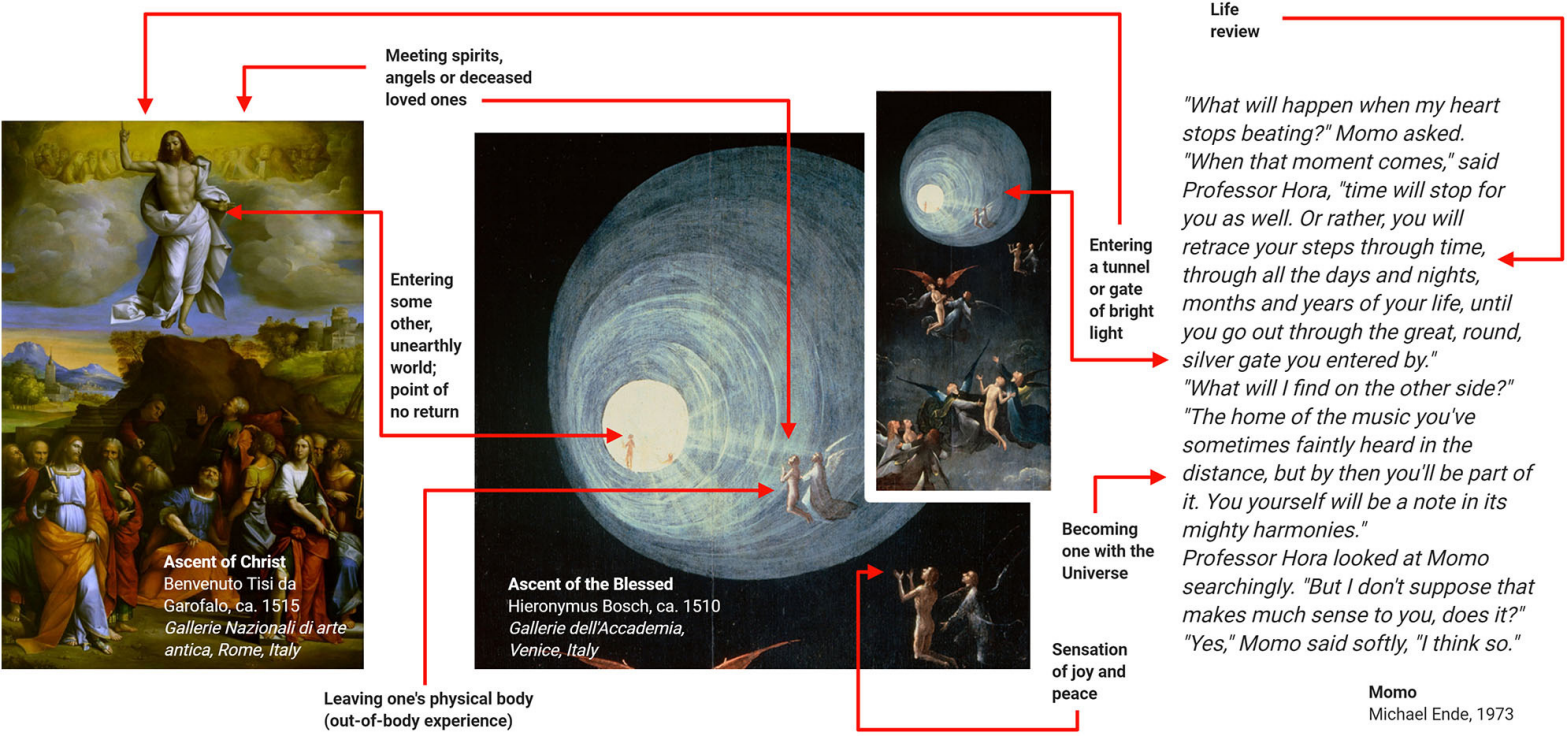
Examples of near-death experiences (NDE) from religion, the visual arts and the literature. These examples suggest that NDE have contributed to various branches of human culture. Adapted with permission from (60).

NDE are conscious perceptual experiences, including self-related emotional, spiritual, and mystical experiences, occurring in close encounters with death or in non-life-threatening situations (59, 60). Typical elements comprise life reviews, out-of-body experiences, entering a tunnel of bright light, feeling peace and joy, becoming one with universe, meeting spirits and entering an unearthly realm (61–63). Accounts of NDE have been collected from all parts of the world, spanning many centuries and various cultural areas including religion, the visual arts, and fictious literature (Figure 7).

**Figure 8:**
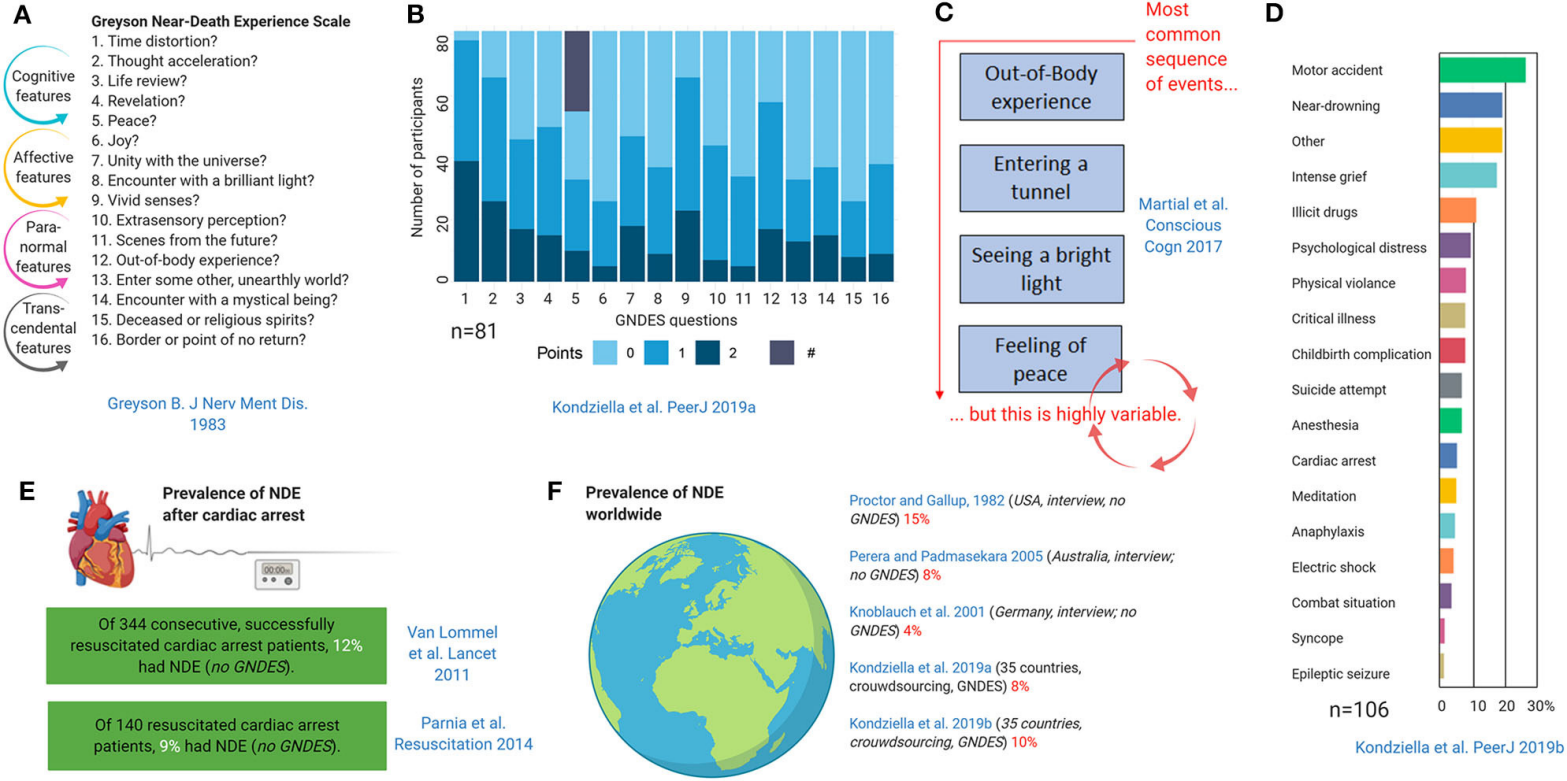
Epidemiology and phenomenology of near-death experiences (NDE) (11, 12, 63, 66–71). The Greyson near-death experience scale (GNDES) is the most often used tool to rate and classify experiences suggestive of NDE **(A)**. An arbitrary cut-off of seven points on the GNDES (maximum 32 points) is considered as indicating a valid NDE, but the distribution of experienced features is highly variable [**(B)**; # denotes participants with NDE associated with negative emotions]. Still, some sequences are more common than others **(C)**. NDE may arise under many different circumstances **(D)**, the perhaps most archetypical being cardiac arrest **(E)**. Prevalence studies using either traditional interview-based or crowdsourcing approaches suggest that NDE are a relatively common, albeit underreported, phenomenon in the general public **(F)**.

Although NDE may have an esoteric flavor, their stereotypy and universality make it likely that there is a biological basis (60). Like other entirely subjective neurological phenomena such as migraine aura or time-space synesthesia, once NDE are systematically described and classified, epidemiologically studies on prevalence and semiology and testing of biological hypotheses become possible (10–12, 64, 65) (Figure 8). Thus, the Greyson NDE scale (66), which consists of 16 questions with a maximum of 32 points and an (arbitrary) cut-off of 7 points for a validated NDE, allows conclusions that the phenomenology of NDE appears to be indistinguishable in life-threatening and non-life-threatening situations (11, 12, 63, 72, 73) and that the sequence of features is highly variable (63). NDE occur in around 9–13% of cardiac arrest survivors (67, 74), and the worldwide prevalence in the general public is around 8–10% (11, 12, 68–70).

Although there is no cerebral lesion associated with NDE, electrical stimulation of and structural lesions at the temporoparietal junction have been linked to out-of-body experiences (9). This makes sense given that in this brain area tactile, proprioceptive, and vestibular inputs are integrated into the perception of personal space, so dysfunction of the temporoparietal junction is believed to lead to disintegration of personal (tactile, proprioceptive) and extra-personal (visual) space, which results in an out-of-body experience (9).

In a remarkably creative attempt to assess the neurochemical underpinnings of NDE, Martial et al. used a text mining approach to search for semantic similarities in 15,000 written consumer reports on 165 psychoactive substances and 625 NDE narratives (10). The substance standing out was ketamine, a NMDAR antagonist. Supporting the importance of NMDAR hypofunction, ketamine is associated with dissociative properties (75); these properties are a psychiatric feature of NMDAR encephalitis (76); and recreational use of ketamine may induce NDE (10, 12).

**Figure 9:**
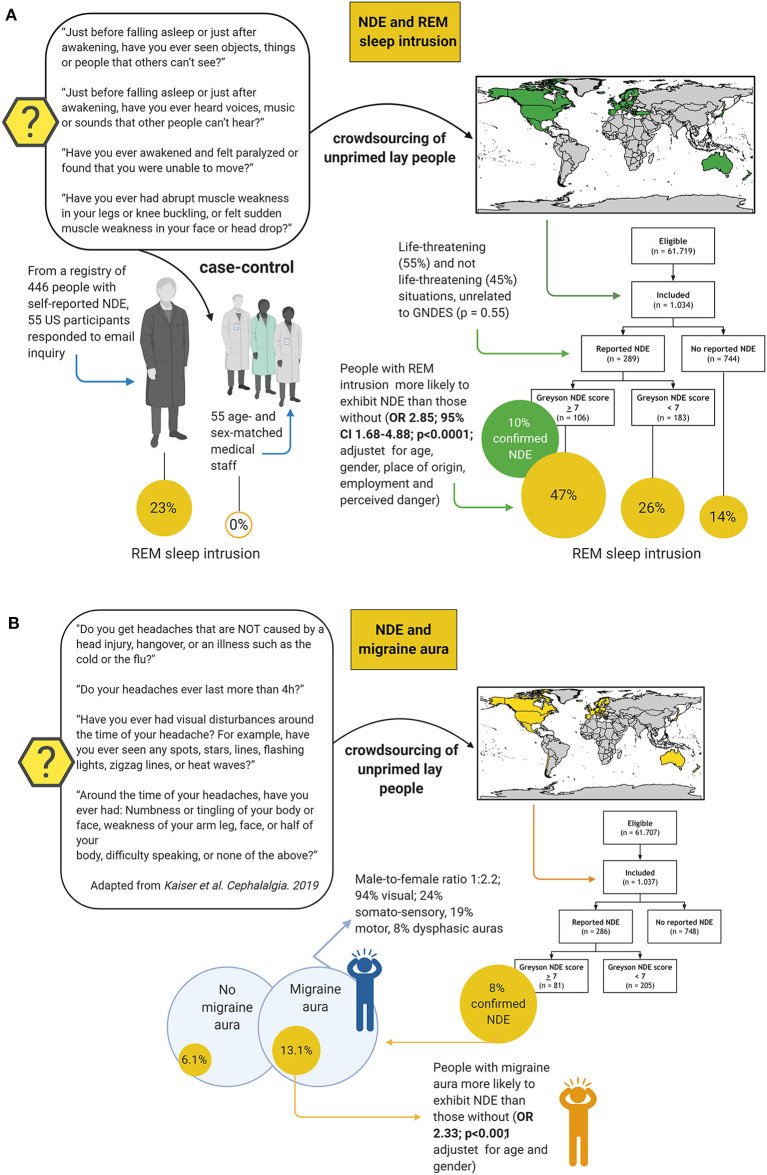
Studies testing rapid eye movement (REM) sleep intrusion and migraine aura as predictors for near-death experiences (NDE). Two studies have shown that REM sleep intrusion into wakefulness is a predictor of NDE **(A)**; while one other has found that this is also the case, albeit perhaps to a somewhat lesser extent, for migraine aura **(B)**. Case-control study on REM sleep intrusion: (77); crowdsourcing study on REM sleep intrusion: (11); crowdsourcing study on migraine aura: (12). CI, confidence interval, OR, odds ratio.

Other biological hypotheses that have been tested include REM sleep intrusion (11, 77) and migraine aura (12) (Figure 9). REM sleep is an attractive candidate mechanism for NDE because it is a natural phenomenon, occurs instantaneously, occurs several times each night in everyone, is associated with dissociative features including muscle atonia and hallucinations, and REM sleep intrusion into wakefulness is a feature of narcolepsy as well as of healthy people (78–80). So far, two studies have investigated the association of NDE with REM sleep (11, 77). In a case-control study, the prevalence of REM sleep intrusion was 23% in a sample of people with NDE and 0% in controls (77). This study was criticized because of its selection bias: Although the groups matched for age and gender, the NDE sample was drawn from people who had taken the initiative to self-register their experience online, whereas the control group consisted of medical staff who most likely was more aware of the implications of the questions related to REM sleep intrusion (81). A crowdsourcing study recruiting over 1,000 unprimed laypeople from 35 countries, however, confirmed an association: While age, gender, place of residence, employment status, and perceived threat did not seem to influence the prevalence of near-death experiences, people with REM intrusion were much more likely to exhibit NDE than those without (OR 2.85, *p* < 0.0001) (Figure 9A). Migraine aura is also an attractive candidate mechanism for NDE because a short-lasting variant of SD is considered the pathophysiological correlate of migraine aura, while terminal SD occurs in humans at the end of life as outlined earlier. Indeed, migraine aura was also a predictor of NDE in another crowdsourcing study of unprimed lay people adjusted for age and gender (OR 2.33, *p* < 0.001) (Figure 9B), but it has not been investigated in more traditional interview-based surveys yet.

**Figure 10:**
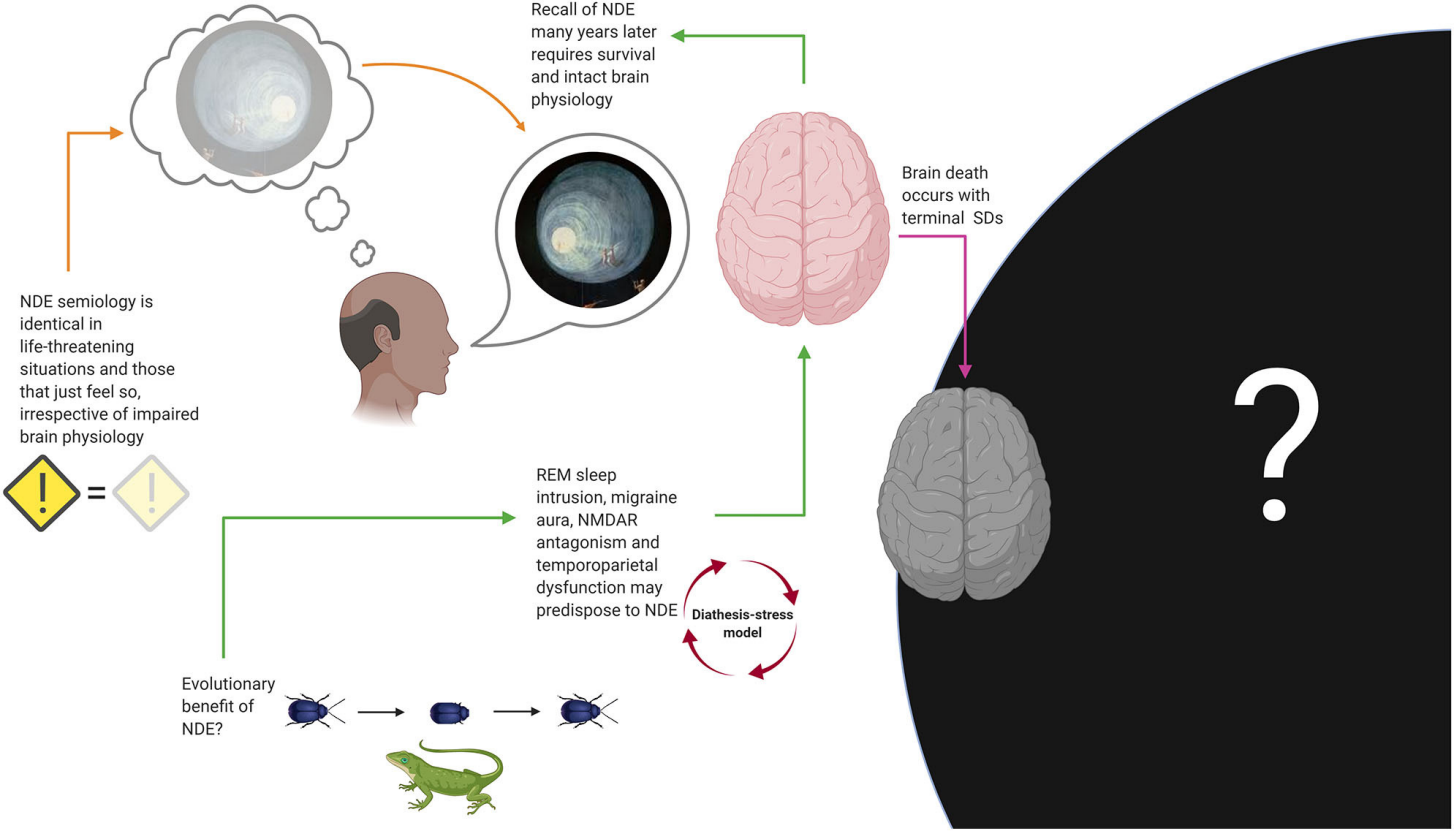
Suggestions for a unifying model of near-death experiences (NDE). Several biological mechanisms, including rapid eye movement (REM) sleep intrusion, N-methyl-D-aspartate receptor (NMDAR) antagonism, temporoparietal cortex dysfunction, and migraine aura, may predispose to NDE, which would be compatible with a diathesis-stress model. Recall of NDE many years later, as well as the fact that NDE associated with true danger are phenomenologically indistinguishable from those associated with situations that objectively lack an element of danger, suggests that normal brain physiology is required to experience and store NDE. Although it seems reasonable to think that some of us will have an experience with features resembling NDE while dying, we will likely never know how common this is. In the same vein, if we presume that a brain is needed to produce consciousness, death should be incompatible with perception of any kind. Thanatosis, also called death-feigning, is an anti-predator strategy and a common phenomenon in the animal kingdom that improves survival fitness and may represent an evolutionary explanation for the occurrence of NDE in humans, although this remains speculation too. SD, spreading depolarization.

Although REM sleep intrusion and migraine aura are strongly associated with NDE (the first perhaps even more so), neither of these two predictors, nor NMDAR hypofunction, and dysfunction of temporoparietal cortex, are sufficient or necessary to explain NDE. Figure 10 shows an attempt to construct a unifying model using the evidence discussed.

According to the hypothesis of Nelson and colleagues, danger (be it real or only perceived) stimulates neuronal pathways that generate REM-associated responses (77, 81). This can be interpreted as a “diathesis-stress model”: An unusually sensitive arousal system (i.e., the diathesis), as evidenced by the experience of REM sleep intrusion, would predispose people to NDE in life-threatening situations and emotional stress (77, 81). Migraine aura, NMDAR antagonism and temporoparietal dysfunction could be understood as contributing factors. This model seems consistent with the fact that the semiology of NDE appears to be identical in situations that are associated with real danger and the possibility for compromised brain physiology (e.g., during resuscitation after cardiac arrest), situations associated with real danger but without evidence of impaired brain physiology (e.g., a near miss traffic accident) and in those that may only just feel threatening but where true danger is absent (e.g., meditation). In any case, recall of NDE many years later requires that the subject has survived without any major brain damage. We can conclude that it seems likely that some of us will experience features of NDE during the process of dying and just before loss of consciousness and immediate death, but we may never know how *many* of us, because for evident reasons no one will ever have the chance to provide this information after they have died. If terminal SD is compatible with the possibility for any conscious percept remains to be determined (12).

Given that NDE is a universal phenomenon that has been preserved in humans for many centuries, the question arises if it serves a specific biological *purpose*. Metaphysical explanations aside, the author hypothesizes that NDE may have an evolutionary benefit. There are countless examples within the animal kingdom that playing dead saves lives (Figure 10). Thanatosis or death-feigning occurs in invertebrates (82–85) and vertebrates (86, 87), including mammals (88, 89) and possibly humans (90), indicating that it is a phylogenetically highly preserved phenomenon. Thanatosis is an anti-predator strategy and the terminal defense response when all other options of fight or flight are futile (91–94). It is characterized by the sudden onset of immobility, with or without loss of tonic muscular activity, and unresponsiveness to external stimuli, while awareness of the environment is fully preserved (92). This is not unlike lucid dreaming and cataplexy which are features of REM sleep intrusion into wakefulness (80) that can occur in NDE (11). Thanatosis increases chances for escape from imminent danger and, being a heritable behavioral trait, it can evolve under natural selection for fitness of survival (95). Although its association with NDE is still entirely speculative, it seems fair to say that panicking in situations of unescapable danger is never a good option for humans either.

## Conclusions

As shown here, neurologists are well-equipped to address the science behind questions about the transition from life to death. Key areas for future research and activities include aligning brain death guidelines across countries, increasing the rate of organ donation by implementing DCD, determining the length of the “hands-off period” prior to organ procurement, improving methods to detect and prevent terminal SDs in intensive care patients with brain injury, and investigating if terminal SDs are compatible with NDE-like features and if thanatosis can be confirmed as the evolutionary origin of NDE. Moreover, in the perhaps not so distant future, neurologists will be consulted about their opinions on ethical considerations (96, 97) related to attempts of brain resuscitation (8). In a highly publicized experiment, Vrselja et al. recently showed they were able to preserve cytoarchitecture, to attenuate neuronal cell death, and to restore vasculature and glial cell responses as well as active cerebral metabolism and synaptic activity in the pig brain 4 h after death (8). Although recovery of consciousness was ruled out by lack of global electrocorticographic activity, this shows that within the neurology of the dying brain, boundaries are being pushed just like in any other area of the clinical neurosciences.

## Author Contributions

The author confirms being the sole contributor of this work and has approved it for publication.

## Conflict of Interest

The author declares that the research was conducted in the absence of any commercial or financial relationships that could be construed as a potential conflict of interest.

## Footnotes

^1^https://www.organdonor.gov/statistics-stories/statistics.html

^2^http://www.ont.es/publicaciones/Documents/NewsleTTER%202018%20final%20CE.pdf
